# Treatment of specific macrovascular beds in patients with diabetes mellitus

**DOI:** 10.1186/1750-4732-4-5

**Published:** 2010-08-11

**Authors:** Allison M Petznick, Jay H Shubrook

**Affiliations:** 1 Diabetes and Endocrine Center, Ohio University College of Osteopathic Medicine, Athens, OH 45701, USA

## Abstract

In 2007, over 23 million people had diabetes in the United States and death from cardiovascular disease is estimated to occur in 80% of those Americans. Risk factor reduction is the most important therapy for primary and secondary prevention of macrovascular disease in patients with and without diabetes mellitus. Despite this, presentation and response to therapy is often different for patients with diabetes compared to their non-diabetic counterparts. This paper will review the current targets for therapy of cardiovascular disease, peripheral vascular disease, and cerebrovascular disease in patients with diabetes.

## 

In 2007, 23.6 million people in the United States had diabetes [1]. Currently, death from cardiovascular disease (CVD) is estimated to occur in 80% or 18.4 million of those Americans [1]. Many patients haven't even been diagnosed with type 2 diabetes until a cardiovascular event has already occurred. Approximately 30% of patients with an acute MI are newly diagnosed with type 2 diabetes prior to hospital discharge [2]. Diabetes already costs the US population 174 billion dollars a year in direct and indirect expenses [1]. It has been estimated that 1 in 5 US Medicare dollars are spent on the person with diabetes [3]. Diabetes, its projected expansion, and its complications could cripple the economy of our health care system. Unfortunately, we are currently only observing the tip of the iceburg.

This paper will review the current targets for therapy in risk factor reduction as well as the approach to screening and therapy of cardiovascular disease, peripheral vascular disease, and cerebrovascular disease in patients with diabetes.

## Current Risk Factor Reduction Targets

Risk factor reduction is the most important therapy for primary and secondary prevention of macrovascular disease in patients with diabetes. The greater the number of risk factors, the greater the overall risk of macrovascular disease [4]. Risk factor reduction includes lifestyle management, blood pressure control, lipid management, glucose control, tobacco cessation, and antiplatelet therapy.

The most effective approach to decreasing cardiovascular mortality is to address hypertension first, then hyperlipidemia, and finally glucose control [5]. Recommended treatment goals for these risk factors are listed in table 1[6]. The independent role of intensive glucose control in the reduction of macrovascular disease has never been proven in a large prospective randomized controlled trial. The goals for A1c were established based on evidence linking a reduction in microvascular, not macrovascular, outcomes to A1c values of < 7% [7-9].

**Table 1:** ADA CV reduction targets in patients with diabetes [6]

	Goal
**A1c**	< 7.0%

**Blood pressure (ADA)**	< 130/80 mmHg

**LDL-C (ADA)**	< 100 mg/dl (very high risk) < 70 mg/dl (highest risk)

**HDL-C (ADA)**	> 40 mg/dl (men) > 50 mg/dl (women)

**Triglyceride (ADA)**	< 150 mg/dl

## Summary Approach to Specific Vascular Beds: Cardiovascular disease

The ADA recommends cardiovascular screening in select patients with diabetes despite the lack of evidence for improved outcomes. Screening is recommended in patients with typical or atypical angina, abnormal EKG suggestive of ischemia or infarct, and age > 35 years with sedentary lifestyle and planning to start a vigorous exercise program. This recommendation is based upon expert opinion.

The San Antonio Heart Study demonstrated that the risk for a cardiovascular event was similar in patients with diabetes and no history of CVD as in patients without diabetes and a history of CVD [10]. As a result, many organizations have designated diabetes as a cardiovascular risk equivalent. Despite this increased risk there has been no evidence that screening for disease in patients with diabetes improves morbidity or mortality. This is believed to be due to the type of atherosclerotic lesion that patients with diabetes have. Most ischemic events originate from an atherosclerotic plaque that is not obstructive and therefore would not be detected by testing. These events are believed to be due to endothelial dysfunction and decreased nitric oxide production, leading to a state of hyper-constriction as well as an increase in unstable plaque and plaque rupture [11].

Silent ischemia is more common in patients with diabetes and there are no symptoms suggestive of an event. Studies have suggested that silent ischemia is present in up to 20-50% of patients with diabetes [12]. The DIAD study looked at over 1,000 patients with type 2 diabetes without a history of CAD and without any cardiac symptoms. The aim of the study was to look for predictors of silent ischemia in people with type 2 diabetes. The initial results of this study found that 22% of patients had silent ischemia, and the strongest predictors for abnormality were cardiac autonomic neuropathy, male sex, and diabetes duration. The so called traditional risk factors were not noted to be predictors [13]. The five year follow up data is not yet available.

If screening is necessary, the test of choice is the exercise stress test. A plain stress test without imaging may be appropriate for male patients with a normal EKG, good exercise tolerability, and asymptomatic patients without a history of a cardiovascular event. There is less validity in this test when women and higher risk patients are being evaluated. The addition of imaging with single photon emission computed tomography (SPECT) increases the sensitivity and specificity of this screening tool. The sensitivity and specificity of the SPECT imaging stress test is about 88% and 75% respectively [14]. Other powerful predictors of prognosis can be attained from the stress test in addition to EKG changes and perfusion defects. These include a poor exercise capacity, exercise induced angina, low or a fall in peak blood pressure, reduced heart rate recovery at 1-2 minutes post exercise, and ventricular arrhythmias [14].

Screening for subclinical atherosclerosis has been suggested as a possible tool to assess cardiovascular risk. This includes coronary artery calcium (CAC) and carotid intima media thickness (CIMT). A low CAC or CIMT is very predictive of a low risk for a cardiovascular event. However, studies have reported evidence of subclinical atherosclerosis in 60-70% of patients with diabetes [14]. These scores are a measure of atherosclerotic burden but do not measure the severity or vulnerability of plaque. Therefore, the utility of these tests in changing prognosis or therapy is minimal but may be of some usefulness in certain patients with intermediate risk.

The guidelines for therapy in patients with a previous cardiovascular event are straightforward. A beta blocker, angiotensin-converting enzyme inhibtor (ACE) or angiotensin receptor blocker (ARB), statin therapy, and anticoagulation with either aspirin or clopidogrel are recommended [15]. However, treatment for asymptomatic cardiovascular disease is controversial. There have been multiple studies questioning the most effective approach for patients with stable and unstable CAD. Table 2 gives a brief summary of outcome data for some of the major studies.

**Table 2:** Outcome data from the COURAGE, BARI, and BARI 2D Trials [16-18]

Trial	Outcome	PCI	CABG	MT
**COURAGE**	Death from any cause/nonfatal MI*	19%		18.5%

	Angina free status	74%		72%

**BARI**	Survival*	71%	73.5%	

	Survival (Diabetes subgroup)	45.5%	57.8%	

**BARI 2D**	Survival*	88.3%		87.8%

* Not statistically significant

The Clinical Outcomes Utilizing Revascularization and Aggressive Drug Evaluation (COURAGE) trial enrolled over 2,000 patients with asymptomatic angiographical evidence of CAD [16]. No statistical difference in the primary outcome (death from any cause and nonfatal MI) or the secondary outcome (death, stroke, MI) was noted between percutaneous coronary intervention (PCI) with optimal medical therapy (MT) versus MT alone (p = 0.62). There was a slight improvement in angina free status in the PCI plus MT group (74%) versus the MT only group (72%) (p = 0.35). One limitation to this study is that drug-eluting stents were not available for use at the time of the study, and as a result bare metal stents were almost exclusively used. This trial suggests that MT may be just as effective as PCI in prevention of death and nonfatal MI in patients with stable CAD.

The Bypass Angioplasty Revascularization Investigation (BARI) trial evaluated the effectiveness of PCI versus coronary artery bypass grafting (CABG) in over 1,800 patients with symptomatic multivessel CAD [17]. The primary outcome of survival was similar in both groups. In a sub group of patients with diabetes and multivessel disease, CABG conferred higher survival rates (57.8%) in comparison to PCI (45.5%) (p = 0.025). This suggests that patients with diabetes and diffuse CAD may have better survival outcomes with CABG.

The Bypass Angioplasty Revascularization Investigation in Type 2 Diabetes (BARI 2D) trial examined over 2,000 patients with type 2 diabetes and angiographical evidence of CAD [18]. There was no difference in the primary outcome of survival between the intervention group (PCI or CABG) versus the MT group (p = 0.97). There was also no difference when prompt revascularization was utilized. There was a statistically significant decrease in cardiovascular events with prompt CABG (22.4%) versus MT (30.5%) (p = 0.01). This was mainly due to the decrease in nonfatal MI with CABG (7.4%) versus MT (14.6%). This trial also looked at the use of insulin sensitizing agents versus insulin provisional therapy with a goal A1c of 7.0%. No statistical significance was seen in the primary outcome between the two different therapies (p = 0.89). A similar A1c goal of 7.0% in the insulin sensitization and 7.5% in the insulin provision group was obtained [18].

## Take Home Points

1. Diabetes is considered a cardiovascular risk equivalent.

2. Subclinical atherosclerosis is present in up to 60-70% of patients with type 2 diabetes.

3. PCI is no more effective than optimal medical therapy in patients with type 2 diabetes and asymptomatic atherosclerosis.

4. Patients with type 2 diabetes and diffuse atherosclerosis may benefit more from CABG than PCI.

## Summary: Approach to Specific Vascular Beds: Peripheral vascular disease

Peripheral arterial disease (PAD) affects 20-30% of patients with diabetes mellitus and is associated with increased coronary heart disease, stroke, and all cause mortality [19]. Risk factors for PAD in patients with diabetes include increasing age, smoking, hypertension, dyslipidemia, neuropathy, and elevated CRP. Typically this involves the femoral-popliteal and tibal vessels and is a more diffuse disease. Over 50% of these patients have asymptomatic disease due to the distal location and associated neuropathy that is usually present. Most will have atypical symptoms which manifest as leg fatigue and slower gait. They may experience intermittent vascular claudication which must be distinguished from neurogenic claudication. Vascular claudication may manifest as pain, cramping, or aching in the legs that is reproducible with walking exercise and goes away with standing and rest. This is different than neurogenic claudication in that the patient needs to sit down for the symptoms to resolve. Patients who get this vascular claudication at rest may develop nonhealing ulcers, or gangrene, which is considered critical limb ischemia and is a medical emergency [20].

**Figure 1:**
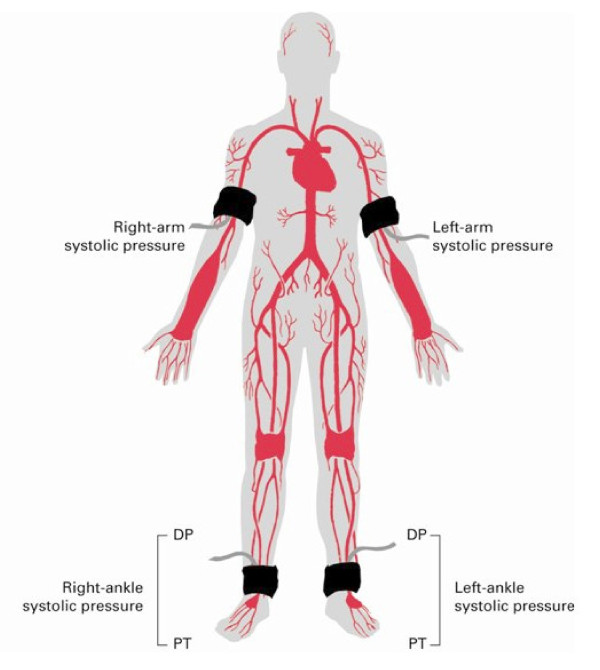
**Ankle brachial index (ABI)**. Blood pressure cuffs are placed on both arms and ankles. Systolic blood pressures are measured at the brachial artery and dorsalis pedis artery with the assistance of an ultrasound Doppler. Two measurements are taken from each arm and leg and then recorded as an average. The ABI is then calculated when the systolic blood pressure in the ankle is divided by the systolic blood pressure in the arm (21).

Due to the vague nature of the symptomatology and the severe burden that PAD places on patients with diabetes, the ADA has recommended a screening ankle brachial index (ABI) in all patients over 50 years of age [6]. This should be repeated every 5 years and should be considered in patients < 50 yrs of age who have other risk factors [20]. The ABI is a diagnostic screening tool where the ratio of systolic blood pressure in the ankle is divided by the systolic blood pressure in the arm (figure 1). This test has a sensitivity of 95% and a specificity of almost 100% [20,21]. A false elevation in ABI may occur due to medial arterial calcification making the arteries poorly compressible. This is usually considered when the ABI is > 1.30 or if PAD is still suspected in a patient with a normal ABI value (table 3).

**Table 3:** ABI values that correspond to severity of PAD [20,23]

	ABI	Typical symptoms
**Normal**	0.91-1.30	Asymptomatic claudication

**Mild**	0.70-0.90	Intermittent claudication

**Moderate**	0.40-0.69	Daily rest pain

**Severe**	< 0.40	Focal tissue necrosis

A pulse volume recording (PVR) is the next test utilized to find the location and severity of the lesion. An exercise treadmill test may be useful to induce the patient's symptoms and to determine pre and post exercise ABI values. These patients will usually have a > 20 mmHg drop in ankle pressure after exercise [20]. Duplex ultrasound, magnetic resonance angiogram (MRA), and xray angiography are then utilized for patients in whom a revascularization procedure is intended.

Therapy for these patients consists of modification of risk factors, a supervised exercise program, pharmacotherapy, and revascularization procedures. Smoking is the most important modifiable risk factor for the progression of PAD [19]. Patients with diabetes have a risk of developing PAD that is four times the general population and patients that smoke and have diabetes have an additional 2.5 fold increased risk [22]. Poor wound healing, increased risk of amputation, and increased mortality has been attributed to smoking. However, smoking cessation has been associated with a 10 year survival rate of 82% versus 46% for patients that continue to smoke [23]. There is no good evidence that treatment of glycemia, hypertension, or hyperlipidemia improves limb outcomes in patients with diabetes and PAD. This is due to lack of studies evaluating the combination of patients with diabetes and PAD. However, based on extrapolation from cardiovascular studies, it is believed that intensive therapy of these conditions will improve outcomes in these patients.

Antiplatelet therapy with aspirin is recommended for all patients with diabetes who have a 10 year estimated cardiovascular risk > 10%, unless there are contraindications to its use. This includes men > 50 years of age and women > 60 years of age who have at least one additional risk factor (family history CVD, hypertension, smoking, dyslipidemia, or albuminuria) [6]. In a sub-analysis of the Clopidogrel versus aspirin in Patients at Risk of Ischemic Events (CAPRIE) trial, clopidogrel in patients with PAD was associated with a 24% risk reduction in vascular events (MI, ischemic stroke, and vascular death) in comparison with aspirin [24]. Despite these results, aspirin is still recommended as first line therapy.

Only 1-2% of patients with peripheral vascular disease will go on to develop critical limb ischemia. The majority of patients will continue with chronic stable claudication. Therefore, most treatment is aimed at improving claudication symptoms and walking distance. Supervised exercise therapy of at least three times a week for three months is associated with improved outcomes and has been shown to be as effective as revascularization therapy [25]. Cilostazol, a phosphodiesterase type III inhibitor, may be useful in symptomatic PAD and is associated with increased maximal walking time, improved functional status, and improved quality of life [19]. Its use is contraindicated in patients with heart failure. Revascularization procedures are indicated in patients that fail conservative therapy, have non-healing ulcerations, gangrene, or critical limb ischemia.

Percutaneous transluminal angioplasty (PTA), with or without stent placement, is best suited for short segment stenosis or large bore vessels while surgical revascularization is better suited for multivessel disease involving smaller and more distant vessels. Guidelines from the American College of Cardiology and the American Heart Association (ACC/AHA) recommend PTA as primary therapy for common and/or external iliac artery stenosis and occlusions with stenting as the primary therapy for those lesions. Stent placement is not recommended in the femoral, popliteal, or tibial arteries due to the high rate of restenosis [26].

## Take Home Points

1. ADA recommends a screening ABI every 5 years in patients with diabetes over the age of 50.

2. Smoking is the most important modifiable risk factor for the progression of PAD.

3. Revascularization procedures are indicated in patients that fail conservative therapy, have non-healing ulcerations, gangrene, or critical limb ischemia.

## Summary: Approach to Specific Vascular Beds: Cerebrovascular disease

Despite no direct relationship between hyperglycemia and stroke incidence, patients with diabetes have a two to four fold increased risk of stroke over the general population [1]. Insulin resistance and the inflammatory cascade have been correlated with an increased stroke incidence [27]. Hyperglycemia has been linked to recurrence of stroke in patients with diabetes that is 2 to 5 times that of patients without diabetes. There is also an increased risk of morbidity and mortality with a 5 year survival rate of only 20% [28]. Ischemia causes the majority of strokes, which are mainly due to occlusion of the small paramedial penetrating arteries that lead to small white matter infarcts, lacunar infarcts. This has also been associated with an increase in stroke related dementia [28].

Risk factors for stroke in patients with diabetes include hypertension, hyperglycemia, hyperlipidemia, tobacco use, metabolic syndrome, hyperuricemia, proteinuria, atrial fibrillation, and other vascular diseases. Hypertension is the single most important risk factor. This risk can be decreased with antihypertensive agents by 30-40%. ACE inhibitors and ARB medications have been shown to be superior to other antihypertensive medications in the prevention of stroke [29-31]. Calcium channel blockers have also shown significant benefit in patients with diabetes [27]. In the Syst-Eur trial, over 4,000 patients > 60 years of age with hypertension were randomized to either nitrendipine plus enalapril and hydrochlorothiazide if needed or placebo. The primary endpoint, composite of fatal and nonfatal stroke, was met with a 42% reduction in the therapeutic arm. This study suggests that the calcium channel blocker, nitrendipine, has an even greater benefit in patients with diabetes. Overall mortality was decreased by 55% in the diabetic population versus 6% in the nondiabetic population [27].

Hyperlipidemia is also important risk factor in the prevention of stroke and statin therapy has been shown to reduce this risk. Studies with statin interventions have shown a 20-50% reduction in stroke [32,33]. Treatment of hyperglycemia in the prevention of stroke is controversial but there was a trend for reduction in stroke risk in the UKPDS. Risk of stroke decreased by 17% with every 1% decrease in A1c [7]. Insulin sensitizers have shown some benefit in the risk of stroke in patients with diabetes. Patients in the UKPDS metformin subgroup found a 42% reduction in stroke risk [8]. Therapy with both of the commercially available PPAR agonists (TZD) have shown improved cerebral hemodynamics and decreased oxidative stress. They are believed to work centrally on the neural and microglial cells, affecting the post stroke prognosis. In the PROactive study, Pioglitazone was associated with a decrease in recurrent stroke by 47% [34].

Intensive glucose therapy in patients with or without diabetes is very important during the acute phase of an ischemic stroke in order to limit the ischemic area. Acute stress hyperglycemia occurs with about 2/3 of ischemic strokes. This provokes anaerobic metabolism, lactic acidosis, and free radical formation, which in turn, leads to lipid peroxidation and cell lysis in the metabolically challenged tissue. There is an area in an acute stroke called the pneumbra. This area has not yet infarcted but is at risk. In patients with hyperglycemia, the infarct extends further into the penumbra leading to an increased area of infarction and a worsening of disability [35]. Imaging studies have shown initial infarct size and progression in hyperglycemic patients [27]. In one study, normalization of glucose was associated with a reduction in mortality by 4.6 times [36].

Interventional therapy has been associated with increased risk in patients with diabetes. Thrombolytics utilized in ischemic stroke are associated with a greater risk of subsequent hemorrhage and patients have less improvement in function than the general population. Atherosclerotic plaque is also more prone to rupture in diabetic patients. This is assumed to be the reason for increased morbidity and mortality with recanalising procedures [28]. This increased risk should be taken into account when utilizing these therapies and determining the risk benefit ratio for patients with diabetes.

## Take Home Points

1. Patients with diabetes have a 5 year survival rate of only 20% after suffering a stroke.

2. Intensive glucose control is associated with a reduction in area of cerebral infarction.

3. Interventional therapy is associated with increased risk in patients with diabetes.

## Summary

As the prevalence of diabetes continues to grow, the prevalence of macrovascular complications will also increase exponentially. There is no evidence that intensive glucose control decreases the risk of these complications. However, multifactorial risk reduction significantly decreases the risk for both primary and secondary prevention.

Silent ischemia in the cardiovascular, peripheral vascular, and cerebrovascular systems are very common in patients with diabetes. Presentation and response to therapy may be different for patients with diabetes compared to their non-diabetic counterparts. Therefore, physicians caring for these patients must have a high index of suspicion and be up to date on the latest evidence for prevention and therapy.

## Competing interests

The authors declare that they have no competing interests.

## Authors' contributions

AP and JS contributed to the research, writing, and review of this manuscript. All authors read and approved the final manuscript.
